# Supplementary material for: Can visual impairment impact your income potential?
Source: PLoS One. 2023 Apr 20;18(4):e0284553. doi: 10.1371/journal.pone.0284553 (PMC10118095; doi:10.1371/journal.pone.0284553)
Supplement: Attachment — Submitted filename: Response to Reviewers.docx [file pone.0284553.s002.docx]

**Point by point response to editor and reviewers**

Dear editor and the reviewers,

We would like to express our profound appreciation to the editor and the reviewers for the valuable comments and suggestions made on our manuscript which were very helpful in revising and improving it.

Please note that the line numbers referred in this document is aligned with the revised manuscript which has track changes.

| **Comments of the Reviewer** | **Comments of the Authors** |
| --- | --- |
| 1. Is the manuscript technically sound, and do the data support the conclusions?  The manuscript must describe a technically sound piece of scientific research with data that supports the conclusions. Experiments must have been conducted rigorously, with appropriate controls, replication, and sample sizes. The conclusions must be drawn appropriately based on the data presented.  **Reviewer #1: Partly**  **Reviewer #2: Partly** | Well noted. Thank you for your comment. Comments have been incorporated in the revised version. |
| 2. Has the statistical analysis been performed appropriately and rigorously?  **Reviewer #1: No**  **Reviewer #2: No** | Duly noted! Thank you! Response well considered and expanded the statistical analysis. |
| 3. Have the authors made all data underlying the findings in their manuscript fully available?  The [PLOS Data policy](http://www.plosone.org/static/policies.action#sharing) requires authors to make all data underlying the findings described in their manuscript fully available without restriction, with rare exception (please refer to the Data Availability Statement in the manuscript PDF file). The data should be provided as part of the manuscript or its supporting information or deposited to a public repository. For example, in addition to summary statistics, the data points behind means, medians and variance measures should be available. If there are restrictions on publicly sharing data—e.g. participant privacy or use of data from a third party—those must be specified.  **Reviewer #1: Yes**  **Reviewer #2: Yes** | Noted with thanks! |
| 4. Is the manuscript presented in an intelligible fashion and written in standard English?  PLOS ONE does not copyedit accepted manuscripts, so the language in submitted articles must be clear, correct, and unambiguous. Any typographical or grammatical errors should be corrected at revision, so please note any specific errors here.  **Reviewer #1: No**  **Reviewer #2: Yes** | Comment well noted with thanks. Language has been improved. |
| **ACADEMIC EDITOR** | |
| **Comment 1** |  |
| Please ensure that your manuscript meets PLOS ONE's style requirements, including those for file naming. | Thank you for your comment. The point has been taken into consideration and kindly note that we have followed the guidelines given and adjusted our manuscript accordingly. |
| **Comment 2** |  |
| During your revisions, please note that a simple title correction is required. Please change the words ' be a drain on' to 'impact': "Can Visual Impairment Impact Your Income Potential?" Please ensure this is updated in the manuscript file and the online submission information. | Noted on this comment made, with many thanks!  The title is corrected as suggested by the editor in the revised manuscript |
| **Comment 3** |  |
| We note that the grant information you provided in the ‘Funding Information’ and ‘Financial Disclosure’ sections do not match.  When you resubmit, please ensure that you provide the correct grant numbers for the awards you received for your study in the ‘Funding Information’ section. | Duly noted on the comment with thanks. The funding information/disclosure is now in the same format as follows,  “This work was supported by the World Bank, Sri Lanka (AHEAD DOR Grant number 09). The funders had no role in study design, data collection and analysis, decision to publish, or preparation of the manuscript” |
| **Comment 4** |  |
| Thank you for stating the following in the Acknowledgments Section of your manuscript:  “Authors acknowledge the contributions of following institutions and people involved. This is an outcome of research project on ‘Quality of Life and Employability potential of Persons with Visual Impairment and Blindness in Sri Lanka’ of Business School of the Sri Lanka Institute of Information Technology, Colombo Sri Lanka. This project is supported by the World Bank funded Accelerating Higher Education Expansion and Development (AHEAD) programme implemented through the Ministry of Higher Education and the University Grants Commission (UGC) of Sri Lanka, which is coordinated by the Operations Monitoring and Support Team (OMST) of the Ministry. The authors also would like to thank Ms. Gayendri Karunarathne for proof-reading and editing this manuscript.”  We note that you have provided funding information that is not currently declared in your Funding Statement. However, funding information should not appear in the Acknowledgments section or other areas of your manuscript. We will only publish funding information present in the Funding Statement section of the online submission form.  Please remove any funding-related text from the manuscript and let us know how you would like to update your Funding Statement. Currently, your Funding Statement reads as follows:  “This work was supported by the World Bank, Sri Lanka (AHEAD DOR Grant number 09). The funders had no role in study design, data collection and analysis, decision to publish, or preparation of the manuscript.”  Please include your amended statements within your cover letter; we will change the online submission form on your behalf. | Well noted and many thanks for notifying about this information. The acknowledgement section mentioned in the revised manuscript is updated as follows,  “Authors acknowledge the contributions of following institutions and people involved. This is an outcome of research project on ‘Quality of Life and Employability potential of Persons with Visual Impairment and Blindness in Sri Lanka’ of Business School of the Sri Lanka Institute of Information Technology, Colombo Sri Lanka under the Accelerating Higher Education Expansion and Development (AHEAD) project in Sri Lanka, which is coordinated by the Operations Monitoring and Support Team (OMST) of the Ministry. The authors also would like to thank Ms. Gayendri Karunarathne for proof-reading and editing this manuscript”.  We have removed all the funding information from the acknowledgement section and other parts of the manuscript as suggested and advised by the editor.  The funding statement will remain as it is. |
| **Comment 5** |  |
| In your Data Availability statement, you have not specified where the minimal data set underlying the results described in your manuscript can be found. PLOS defines a study's minimal data set as the underlying data used to reach the conclusions drawn in the manuscript and any additional data required to replicate the reported study findings in their entirety. All PLOS journals require that the minimal data set be made fully available. For more information about our data policy, please see http://journals.plos.org/plosone/s/data-availability.  Upon re-submitting your revised manuscript, please upload your study’s minimal underlying data set as either Supporting Information files or to a stable, public repository and include the relevant URLs, DOIs, or accession numbers within your revised cover letter. For a list of acceptable repositories, please see http://journals.plos.org/plosone/s/data-availability#loc-recommended-repositories. Any potentially identifying patient information must be fully anonymized.  Important: If there are ethical or legal restrictions to sharing your data publicly, please explain these restrictions in detail. Please see our guidelines for more information on what we consider unacceptable restrictions to publicly sharing data: http://journals.plos.org/plosone/s/data-availability#loc-unacceptable-data-access-restrictions. Note that it is not acceptable for the authors to be the sole named individuals responsible for ensuring data access.  We will update your Data Availability statement to reflect the information you provide in your cover letter. | Well noted and many thanks for notifying about this information. The minimal data set of the study will be submitted along with the revised manuscript under S1 Appendix. |
| **Comment 6** |  |
| We note that you have indicated that data from this study are available upon request. PLOS only allows data to be available upon request if there are legal or ethical restrictions on sharing data publicly. For more information on unacceptable data access restrictions, please see http://journals.plos.org/plosone/s/data-availability#loc-unacceptable-data-access-restrictions.  In your revised cover letter, please address the following prompts: | Well noted and many thanks for notifying about this information. The authors have decided to publicly share the data without legal or ethical restrictions. |
| **Comment 6: 1** |  |
| If there are ethical or legal restrictions on sharing a de-identified data set, please explain them in detail (e.g., data contain potentially sensitive information, data are owned by a third-party organization, etc.) and who has imposed them (e.g., an ethics committee). Please also provide contact information for a data access committee, ethics committee, or other institutional body to which data requests may be sent. | Thank you for the information. The authors have decided to share the data set without any hesitation with the journal. |
| **Comment 6: 2** |  |
| If there are no restrictions, please upload the minimal anonymized data set necessary to replicate your study findings as either Supporting Information files or to a stable, public repository and provide us with the relevant URLs, DOIs, or accession numbers. For a list of acceptable repositories, please see <http://journals.plos.org/plosone/s/data-availability#loc-recommended-repositories>.  We will update your Data Availability statement on your behalf to reflect the information you provide. | Well noted. The minimal anonymized data set of the study will be uploaded. |
| **Comment 7** |  |
| We note that [Figure 1] in your submission contain [map/satellite] images which may be copyrighted. All PLOS content is published under the Creative Commons Attribution License (CC BY 4.0), which means that the manuscript, images, and Supporting Information files will be freely available online, and any third party is permitted to access, download, copy, distribute, and use these materials in any way, even commercially, with proper attribution. For these reasons, we cannot publish previously copyrighted maps or satellite images created using proprietary data, such as Google software (Google Maps, Street View, and Earth). For more information, see our copyright guidelines: http://journals.plos.org/plosone/s/licenses-and-copyright.  We require you to either (1) present written permission from the copyright holder to publish these figures specifically under the CC BY 4.0 license, or (2) remove the figures from your submission: | Thank you very much for indicating this fact. Kindly note that Figure 01 is an author demonstrated diagram through the Geographic Information System Mapping. Therefore, this submission does not contain any copyrighted images. The following statement is added to the manuscript from line number 127 to 129 in page number 7 under the materials and methods section.  …. “These three locations are located in *North-western, Southern and Northern* provinces, respectively, as shown in Figure 1, drawn using Geographic Information System (GIS) Software”. |
| **Comment 7:1** |  |
| You may seek permission from the original copyright holder of Figure 1 to publish the content specifically under the CC BY 4.0 license.  We recommend that you contact the original copyright holder with the Content Permission Form (http://journals.plos.org/plosone/s/file?id=7c09/content-permission-form.pdf) and the following text:  “I request permission for the open-access journal PLOS ONE to publish XXX under the Creative Commons Attribution License (CCAL) CC BY 4.0 (http://creativecommons.org/licenses/by/4.0/). Please be aware that this license allows unrestricted use and distribution, even commercially, by third parties. Please reply and provide explicit written permission to publish XXX under a CC BY license and complete the attached form.”  Please upload the completed Content Permission Form or other proof of granted permissions as an ""Other"" file with your submission.  In the figure caption of the copyrighted figure, please include the following text: “Reprinted from [ref] under a CC BY license, with permission from [name of publisher], original copyright [original copyright year].” | Thank you very much and well noted. The response to this comment is given in comment 7. |
| **Comment 7:2** |  |
| If you are unable to obtain permission from the original copyright holder to publish these figures under the CC BY 4.0 license or if the copyright holder’s requirements are incompatible with the CC BY 4.0 license, please either i) remove the figure or ii) supply a replacement figure that complies with the CC BY 4.0 license. Please check copyright information on all replacement figures and update the figure caption with source information. If applicable, please specify in the figure caption text when a figure is similar but not identical to the original image and is therefore for illustrative purposes only. | Thank you very much and well noted. The response to this comment is given in comment 7. |
| **REVIEWER #1** | |
| **General Comment** |  |
| The authors present a paper on income and visual impairment/blindness in a sample of adults from Sri Lanka. The following are some comments for consideration and potential improvement of the manuscript: | Thank you very much for sparing your valuable time on reviewing this manuscript. The comments given are very well appreciated and they have been incorporated accordingly. |
| **Comment 1** |  |
| There were many points in the manuscript where the language used felt awkward or interfered with my ability to understand the points that the authors were trying to convey. Assistance from an outside editor may be helpful in improving the readability and clarity of the manuscript. For example, I am not sure what the following paragraph means:  "From the standpoint of total individuals, it was discovered that persons who suffer from some sort of visual impairment are more vulnerable to be tagged mainly with earning income levels of up to SLRs.10,000 and SLRs.10,000-25,000. Remarkably, it was further identified that most severely impaired persons earn a significant income comparable to the above-mentioned income levels. It can be concluded that persons with severe vision disorders have the probability of earning at least up to an income level of up to SLRs.10,000 and SLRs.10,000 to 25,000 correspondingly." | Thank you for indicating this point for improvement. The paper has been revised thoroughly and in-depth proofreading check has been performed by a linguistic professional.  The following paragraph was strengthened with further elaboration in the revised manuscript in page number 25 from line 50 to 54 as follows,  …….“As per the authors' observations, the majority who suffer from severe visual impairment disorders earn an income in the range of SLRs.10,000-25,000. It can be concluded that even with such disorders, this community has the probability of earning an income to satisfy their daily needs”. |
| **Comment 2** |  |
| In the introduction, more information on prevalence of blindness and visual impairment in Sri Lanka specifically would be appreciated. | Thank you very much and the revised version has intensified the introduction. The information on the “prevalence of blindness and visual impairment in Sri Lanka” are stated in the introduction section from line 81 to 85 in page 5  …..“ The survey conducted by the Government of Sri Lanka (GOSL) in 2016 revealed the prevalence of blindness in Sri Lanka was 1.6% and 15.4%, respectively, concerning severe visual impairment and blind conditions. This rate varies for different provinces, where the highest was from Uva and Southern provinces (2.9% and 0.29%)”. |
| **Comment 3** |  |
| More context for what these income levels mean in the Sri Lankan context (e.g., poverty, lower class, middle class, upper class would be helpful) in contextualizing the results. | Thank you for the insightful comment. The methodology section of the paper has been improved by adding the meaning of income levels in the Sri Lankan context. The detail information is available in Table 1. The explanation starts from line 208 to 212 in page 11 and 12. |
| **Comment 4** |  |
| The qualitative portion of the study (Phase 3) was extremely under-developed in its presentation. Qualitative methodology is rich and complex and yet no information was given on the interview content and methods, the coding methods and approaches for the qualitative data, how inter-coder agreement was assessed, how translation occurred if needed, etc. This makes it more or less impossible to truly understand the rigour or meaningfulness of the Phase 3 results. | Well noted with many thanks for the comment. The authors agree with the reviewer’s comment and the qualitative portion of the study is now developed with the necessary information as suggested by this reviewer. The detailed explanation is available in the revised manuscript from line 157 to 174 in page 9 as follows,  “In phase three of the study, explicit interviews were held through focus group discussions (FGDs) to capture the factors affecting the quality of life of persons with VIB. Eleven individuals (four females and seven females) were purposively selected to represent the VIB community. All these participants were from various socio-economic backgrounds and distinct cadres in the society, including lawyers, undergraduates, academicians, entrepreneurs, development officers and committee members from diverse VIB voluntary organisations. The selected sample includes VIB individuals from different age categories ranging from 20 to 60 years living in rural and urban areas of the country. Their detailed information is not disclosed due to ethical reasons and privacy. The FGDs were held online through zoom platform using two languages (Sinhala and English) based on their fluency. The interviews lasted on average 3 hours and 40 minutes. All these discussions were recorded with participants’ prior consent and manually transcribed for analytical purposes”. |
| **REVIEWER #2** | |
| **General Comment** |  |
| I would like to thank all authors for their effort on producing this manuscript. However, I have some major concerns. | Thank you very much for reviewing this paper and we have addressed the valuable comments given in the revised manuscript. |
| **Comment 1** |  |
| Authors have been mentioned in the abstract "Due to this excruciating elevated rate on medication and treatments to cure the optical concerns, the visual disorders of people keep rising day by day." I have my doubts on this point. Many different eye conditions can cause low vision, but the most common causes are: Age-related macular degeneration (AMD), Cataracts.  Diabetic retinopathy (a condition that can cause vision loss in people with diabetes). It’s mainly modifiable and non-modifiable risk factors associated with NCDs. | Thank you for the valuable comment! We agree with the point raised by this reviewer. Many eye conditions can cause low vision as you highlighted like AMD, Cataracts and Diabetic retinopathy.  But even with such reasons one can increase his/her low vision issues, when there is a higher price rate on medication and treatments to cure the optical concerns.  This was what we wanted to emphasise from that sentence. However, since the reviewer had a doubt, and we amended the sentence and it is available in the revised manuscript from line number 36 to 39 in page number 3 as follows,  “Different eye conditions can cause low vision. Apart from all other reasons, due to the unbearable higher price rates on medication and treatments, people's visual disorders keep rising daily keep rising daily.” |
| **Comment 2** |  |
| The objective of this study is to "identify the impact of different levels of vision and socio-economic characteristics towards income of people". I am not very clear about this point whether authors have demonstrated the relevant results. Regression analysis was mainly conducted different Income levels as dependent variable. I am unable to find out confounding effect on socioeconomic characteristics towards impaired vision and income. | Thank you for the comment. As per the objective of the study, we only discovered whether there is an effect from vision impairment and socio-economic characteristics towards income.  To further clarify, this study does not find the confounding effect on socio-economic characteristics towards impaired vision and income as it goes beyond the objective of this study.  In the results and discussion section, under phase two section from line 284 to 295 in page number 16 explains the mediating effect of vision levels and socio-economic status on income levels of this community. |
| **Comment 3** |  |
| Authors have mentioned "Majority of severely impaired individuals are more likely to earn a lower rate of income leading them to have a degraded quality of life." It is not clear how quality of life has been measured. | Whilst thanking for the comment, we would like to highlight that there is no specific tool to measure the quality-of-life dimension. Since the reviewer has a doubt on the clarity of the quality-of-life dimension, we would like to bring out this valuable comment into consideration. This part is added to the results and discussion section under the phase three in page 26 from line 72 to 77 in the revised manuscript as follows,  ….“The World Health Organization (WHO) defines Quality of Life “as an individual’s perception of their position in life in the context of the culture and value systems in which they live and concerning their goals, expectations, standards and concerns” [28, p.3]. It relates to life satisfaction, including everything from physical health, family, education, employment, wealth, safety, security to freedom, religious beliefs, and the environment…” |
| **Comment 4** |  |
| To my understanding visual impairment has always direct effect/impact on income and quality of life. The different visual impairment levels could have different levels of quality-of-life values/levels. Are there any socio-economic characteristics which significantly associated with these different stages. This can be very important for policy decisions. | Thank you for indicating this point for enhancement. As per the objective of the study, we investigated the impact of **socio-economic characteristics** and **vision levels** **towards** **income**. For this study, we didn’t find the association of how socio-economic characteristics are associated with different impairment stages.  Therefore, as per the reviewer’s valuable suggestion, to identify how socio-economic characteristics associate with different impairment levels, we will be conducting another study. It is with great thanks; we would like to incorporate this significant comment into consideration for our next study which is currently under the drafting process. |
| **Comment 5** |  |
| If possible, performing a dimension reduction methods and path analysis will help to assess the hypothesis on above mention points. I believe identifying few unique risk factors which can improved in a developing country with minimal financial input will be more benefited to the community and practically applicable. | Thank you very much for indicating this point for development. Currently we are in the process of collecting an island wide data collection from the VIB community of Sri Lanka which will be completed by the year 2024. We will be incorporating a larger sample size for the next study and for that we are planning to apply the recommended dimension reduction method and path analysis.  We highly believe that it can be helpful to identify the unique risk factors for the development of the country. |
