# Supplementary material for: Can visual impairment impact your income potential?
Source: PLoS One. 2023 Apr 20;18(4):e0284553. doi: 10.1371/journal.pone.0284553 (PMC10118095; doi:10.1371/journal.pone.0284553)
Supplement: Attachment — Submitted filename: Response to Reviewers.docx [file pone.0284553.s003.docx]

**Point by point responses to reviewers**

Dear Reviewer,

We would like to convey our sincere gratitude to the editor and the reviewers for your insightful comments and suggestions on this paper, which helped authors to improve and revise it.

Please take note that this document's line numbers correspond to the amended manuscript's track modifications.

| **Comments of Reviewer 01** | **Comments of the Authors** |
| --- | --- |
| I thank all the authors for there responses. Authors have address all the review comments raised by me. There is a significant improvement and and clarity added to the manuscript. | Thank you very much for the comment and we highly appreciate your time and effort contributed to this manuscript to grow into a success. |
| **Comments of Reviewer 02** | **Comments of the Authors** |
| **Comment 01** | |
| This manuscript needs more extensive editing. | Thank you very much and well noted for raising this point. The comment is well addressed with the below suggested comments in the revised version. |
| **Comment 02** | |
| Introduction is not clear and gives misleading information, objectives not clearly written, methods not described adequately, trying to highlight the statistics made it less understandable, more importantly no clear statement on Ethical clearance for human subject research... | Well noted and thank you for raising these points.  Since the objectives are not clearly written, we have re-structured and is now stated from line number 95 to 98 as follows,  “The objective of the present study is to fill this empirical gap. In doing so, the study can be considered extensive, as it investigates the impact of different levels of vision and other socio-economic characteristics on the income of visually impaired people”  We do agree that the methodology is not described adequately in the version one. In the 2^nd^ round of revision, the same comment was raised, and we incorporated, and the methodology is now in a more understandable and clear flow according to the below structure,   1. Study population and data collection process 2. Statistical analysis with in detail instructions of all types of methodologies used in the study 3. Definitions of each variable 4. Ethical statements   The ethical sentence is further updated (From line number 267 to 276) with the accessed grant numbers to understand the ethical protection of human utilized in the study as follows,  “This study was conducted once the Ethical Clearance Board of Sri Lanka Institute of Information Technology (SLIIT) granted ethical clearance approval. Accordingly, the study objectives were conveyed to respondents prior to commencing the survey and their informed consent was obtained. Participants’ data are secured with a high level of confidentiality and stored under AHEAD Project (DOR 1 HEMS) for Persons with Visual Impairment and Blindness at the SLIIT Business School, Sri Lanka Institute of Information Technology, Malabe, Sri Lanka” |
| **Comment 03** | |
| Do you have evidence to confirm your first sentence in the abstract?  I am not sure about it. On the other hand I am also not sure on which medications you talk about medications which prevent visual impairment and blindness.  Regarding the objective; Everybody know that socio-economic characteristics will adversely affect towards the income of people. So why do you want to study that? | Well noted and many thanks for notifying about this information. Since the first sentence is doubtful, we have changed it to a more globalized setting regarding the persons with visual impairment and blindness from line number 29 to 42 as follows,  “People’s quality of life (QOL) has been disrupted globally in the wake of the pandemic in recent times. This was mainly due to global economic crises fueled by the coronavirus (COVID – 19) and other related factors. Sri Lanka, too, was facing major social and economic constraints in the period 2021 –2022. Thus, all communities island wide have been economically disturbed. Among others, people with Visual Impairment and Blindness (VIB) have been pushed to severely disadvantageous positions, financially and otherwise.”  However, this study does not communicate about any preventive medications. It previously discussed about the medicine price escalation due to high inflation (Dabare et al., 2014)  Therefore, in such a condition, if there is any person who have less vision and can be treatable as per the World Health Organization Statistics, will find it difficult to get the treatments and medicines due to the price escalation.  **(“Globally, at least 2.2 billion people have a near or distance vision impairment. In at least 1 billion – or almost half – of these cases, vision impairment could have been prevented or has yet to be addressed”)**  (<https://www.who.int/news-room/fact-sheets/detail/blindness-and-visual-impairment>)  (Dabare, P.R.L., Wanigatunge, C.A. & Beneragama, B.H. A national survey on availability, price and affordability of selected essential medicines for non-communicable diseases in Sri Lanka. BMC Public Health 14, 817 (2014). <https://doi.org/10.1186/1471-2458-14-817>)    As per your second comment, we admit that everybody knows socio-economic characteristics will adversely affect towards the income of people. Yet, the effect of how persons with visual impairment grapple with the above condition is unknown which led us to conduct this study. Furthermore, there are multiple significances for conducting this kind of a study as follows,   1. It can enlighten the growth of personalized assistance and training programs that deliver the special needs of visually impaired individuals. 2. This study can offer valuable knowledge to employers and policy makers about the economic benefits of hiring and supporting visually impaired workers. 3. It can help to identify and address discrepancies in income and employment prospects between visually impaired individuals and their sighted peers. 4. The study can expand research into the broader societal influences of visual impairment and the significance of addressing this issue from an economic, as well as a social, perspective. 5. This study can further support to challenge stereotypes and misconceptions about the visually impaired individual’s abilities and potentials in the workplace and society more broadly.   As per your valuable comment we have strengthen the significance of the study from line number 99 to 119 follows,  “This research is significant from the existing studies and contributes to the literature in four ways. Firstly, visual impairment and the income level of these victims have become a major issue in the 21st century, in terms of health and economic aspects. In these circumstances, this issue of visual health requires the attention of regulatory authorities such as the government, health sector, policymakers, other organisations and the general public. Secondly, no previous research study has been conducted concerning the area under consideration, addressing the local scenario. According to available information, this study will be the first endeavour of this kind of econometric research study covering the impact of levels of vision and other socio-economic characteristics towards the income of visually impaired and blind persons based in Sri Lanka. Thirdly, according to the World Health Organization (WHO), over 2.2 billion people suffered from visual disorders worldwide; over 90% of them were from low and middle-income countries, which is the main cause of blindness in Asia and Africa [2]. Therefore, after completing this study, the findings can provide valuable insights to the GOSL for formulating strategies and policies. The findings will also benefit the healthcare sector and policymakers aiming to recover from the setback encountered by the COVID-19 pandemic and revive the economy and QOL of visually impaired people. In this regard, the health sector could utilise these data to spread awareness and expand its healthcare facilities regarding this pandemic, especially among individuals with VIB who are badly affected by the crisis.” |
| **Comment 04** | |
| I recommend getting help from a public health specialist and ophthalmologist to write this article and need more concise writing. Please get help from English editing services as well. | Noted with many thanks for these two valuable suggestions!  This study comes as a sub project of the AHEAD research project for persons with visual impairment and blindness. Therefore, prior to writing this article, the data collection questionnaires for quantitative and qualitative investigations, was reviewed and finalized, with medical consultations and ophthalmologists and also the study data was further validated by an entire team consisting different specialist from different areas.  Furthermore, for the second comment, the authors would like to appreciate your valuable suggestion. The paper has been revised thoroughly and in-depth proofreading check has been performed by a linguistic professional. |
| **Comment 05** | |
| This sentence cannot be clearly understood, “The survey conducted by  the Government of Sri Lanka (GOSL) in 2016 revealed the prevalence of blindness in Sri  Lanka was 1.6% and 15.4%, respectively, concerning severe visual impairment and blind  conditions.” | Thank you very much for indicating this point for improvement. The following sentence was used to strengthened with further elaboration in the revised manuscript in page number 04 from line 77 to 82 as follows,  “The survey conducted by the VISION-2020 national workshop programme by the Government of Sri Lanka (GOSL) in 2016 in collaboration with the International Agency for the Prevention of Blindness (IAPB) South East Asia revealed the prevalence of blindness in Sri Lanka was 1.6% and 15.4%, respectively, concerning severe visual impairment and blindness”. |
| **Comment 6** | |
| Blind conditions = blindness | Duly noted and thank you very much for the suggestion. The suggested comment was well addressed in the revised manuscript in page 4 in line number 82 as follows,  “The survey conducted by the VISION-2020 national workshop programme by the Government of Sri Lanka (GOSL) in 2016 in collaboration with the International Agency for the Prevention of Blindness (IAPB) South East Asia revealed the prevalence of blindness in Sri Lanka was 1.6% and 15.4%, respectively, concerning severe visual impairment and blindness”. |
| **Comment 07** | |
| What do you want to say here ? “The survey conducted by  the Government of Sri Lanka (GOSL) in 2016 revealed the prevalence of blindness in Sri  Lanka was 1.6% and 15.4%, respectively, concerning severe visual impairment and blind  Conditions” | Noted with thanks! As per the suggestion given in the second revision by reviewer 01, we included the recent survey statistics of persons with visual impairment and blindness in the Sri Lankan perspective conducted by the vision 2020 program to show the prevalence of these individuals. That is the reason behind including this statement. However, since the statement had some confusions in the previous comment, we revised it and is shown in the above comments 05 and 06. |
| **Comment 08** | |
| Have you got the clearance from an ethical review committee as this study involves human subjects? It is not clear in your statements that you have got is. If yes give the reference numbers and details. | Well noted and thank you very much for the comment. This research paper is based on a specific study under the World Bank funded comprehensive research project entitled quality of life and employability potential of persons with visual impairment and blindness in Sri Lanka. The ethical clearance application was submitted to the Dean – Postgraduate Studies and Research and Chairman of the Ethics Committee of Sri Lanka Institute of Information Technology on 16^th^ March 2021. The approval of the ethics committee was obtained on December 5^th^, 2021. Accordingly, all research activities were conducted strictly under ethical guidelines. This ethical clearance refers to quantitative and qualitative data collections from the persons who are visually challenged and blind in Sri Lanka.  The ethical clearance paragraph is further updated (line number 267 to 276) in page number 14 as follows,  “This study was conducted once the Ethical Clearance Board of Sri Lanka Institute of Information Technology (SLIIT) granted ethical clearance approval. Accordingly, the study objectives were conveyed to respondents prior to commencing the survey and their informed consent was obtained. Participants’ data are secured with a high level of confidentiality and stored under AHEAD Project (DOR 1 HEMS) for Persons with Visual Impairment and Blindness at the SLIIT Business School, Sri Lanka Institute of Information Technology, Malabe, Sri Lanka” |
| **Comment 09** | |
| The readers are not in a position to understand the statistics. Kindly make it clear and simple. | Thank you for raising this information. As per your valuable suggestion, in order to make the statistics more precise and simpler, the authors incorporated detailed information about the methodology used to investigate the impact of the variables in the revised manuscript and we also incorporated textbooks and different articles to further understand the statistics  in page number 10 from line 193 to 204 as follows,  “Before conducting the Ordered probit regression model, it is vital to contemplate three main considerations as to whether the objective fits with the regression model [10, 13-17]. The three main considerations are model specification, model building, and model diagnosis check. The model specifications allow the researchers to identify the dependent and independent variables identified as income and levels of vision along with other socio-economic factors such as age, gender, marital status, employability, and education. The model building allows the researchers to specify the process to build the equation concerning the variables of the present research study; further, it provides the pathway to explain the variation between income (Dependent variable) and levels of vision along with other socio-economic characteristics (Independent variables)….” |
